# Design and Testing of Vector-Producing HEK293T Cells Bearing a Genomic Deletion of the SV40 T Antigen Coding Region

**DOI:** 10.1016/j.omtm.2020.07.006

**Published:** 2020-07-09

**Authors:** Dahae Hailey Bae, Michael Marino, Brian Iaffaldano, Sydney Fenstermaker, Sandra Afione, Takele Argaw, Jacob McCright, Anna Kwilas, John A. Chiorini, Andrew E. Timmons, Jakob Reiser

**Affiliations:** 1 Division of Cellular and Gene Therapies, Center for Biologics Evaluation and Research, US Food and Drug Administration (FDA), Silver Spring, MD, USA; 2 Office of Biostatistics and Epidemiology, Center for Biologics Evaluation and Research, FDA, Silver Spring, MD, USA; 3 AAV Biology Section, National Institute of Dental and Craniofacial Research, NIH, Bethesda, MD, USA

**Keywords:** Lentivirus, AAV, vector manufacturing, HEK293T cells, SV40 T antigen, CRISPR/Cas9

## Abstract

The use of the human embryonic kidney (HEK) 293T cell line to manufacture vectors for *in vivo* applications raises safety concerns due to the presence of SV40 T antigen-encoding sequences. We used CRISPR-Cas9 genome editing to remove the SV40 T antigen-encoding sequences from HEK293T cells by transfecting them with a recombinant plasmid expressing Cas9 and two distinct single guide RNAs (sgRNAs) corresponding to the beginning and end of the T antigen coding region. Cell clones lacking T antigen-encoding sequences were identified using PCR. Whole-genome (WG) and targeted locus amplification (TLA) sequencing of the parental HEK293T cell line revealed multiple SV40 T antigen-encoding sequences replacing cellular sequences on chromosome 3. The putative T antigen null clones demonstrated a loss of sequence reads mapping to T antigen-encoding sequences. Western blot analysis of cell extracts prepared from the T antigen null clones confirmed that the SV40 large and small T antigen proteins were absent. Lentiviral vectors produced using the T antigen null clones exhibited titers up to 1.5 × 10^7^ transducing units (TU)/mL, while the titers obtained from the parent HEK293T cell line were up to 4 × 10^7^ TU/mL. The capacity of the T antigen-negative cells to produce high titer adeno-associated virus (AAV) vectors was also evaluated. The results obtained revealed that the lack of T antigen sequences did not impact AAV vector titers.

## Graphical Abstract

**Figure:**
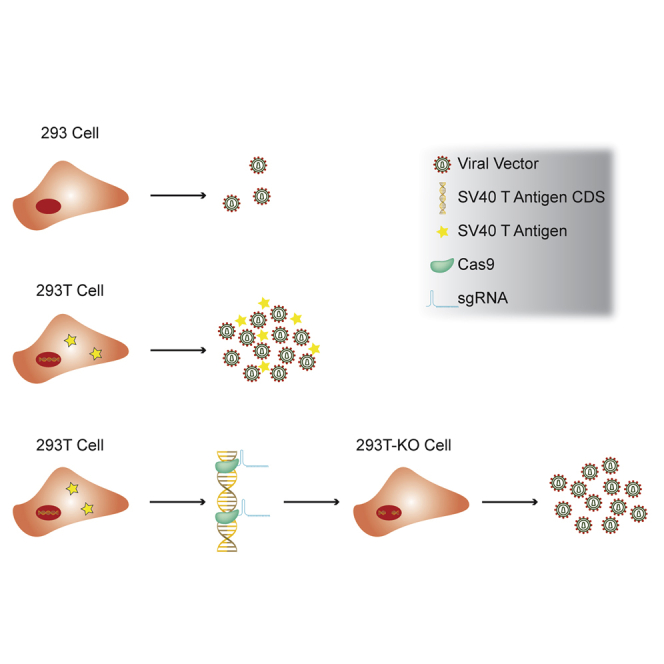

## Teaser

The production of clinical-grade gene therapy vectors requires removal of process and product-derived impurities. In this work, we engineered the human embryonic kidney (HEK) 293T cell line to eliminate SV40 T antigen-related sequences. Despite the absence of the SV40 T antigen, we found that T antigen knockout cells retained the high-titer phenotype of HEK293T cells for lentiviral and AAV2 vector production.

## Introduction

Lentiviral vectors are typically produced using the human embryonic kidney (HEK) 293T cell line. Compared to the parental HEK293 cell line, lentiviral vector titers obtained using HEK293T cells are higher.^1^^,^^2^ The original HEK293 cell line was derived by transformation of a human primary embryonic kidney cell line using mechanically sheared DNA of adenovirus type 5 (Ad5).^3^ A 4-kb Ad5 DNA fragment encoding the E1A/E1B proteins was later shown to have integrated into chromosome 19, resulting in transformation.^4^

HEK293T cells were originally generated by stably transfecting HEK293 cells with a plasmid expressing a temperature-sensitive version of the SV40 large T antigen.^5^^,^^6^ The SV40 large T antigen is known to be capable of transforming human and rodent cells *in vitro* and *in vivo*. Also SV40-transformed human cells are able to produce tumors when administered to nude mice.^7^^,^^8^ Therefore, the presence of residual SV40 T antigen protein and/or T antigen-encoding nucleotide sequences in viral vector lots prepared using the HEK293T cell line poses a significant safety concern.^2^ To mitigate this concern, we engineered HEK293T cells to remove SV40 T antigen-encoding sequences using a CRISPR-Cas9 deletion approach. Individual cell clones were genotyped by PCR to confirm the absence of T antigen-encoding sequences. The putative knockout cell clones obtained were further validated by western blot analysis. Next-generation sequencing (NGS) of SV40 T antigen-negative cell clones, as well as the parental HEK293T cell line, confirmed the presence of deletions within the SV40 large T antigen coding sequence. In addition, targeted locus amplification (TLA) sequencing^9^^,^^10^ allowed further characterization of the integration site of the pRTAK SV40 T antigen plasmid originally used to derive the HEK293T cell line.^11^ This analysis revealed multiple plasmid copies within a 550-kb deletion of cellular sequences on chromosome 3.

Despite the absence of the SV40 T antigen, we found that T antigen knockout clones retained the high-titer phenotype of HEK293T cells for lentiviral vector production. When producing adeno-associated virus (AAV)2 vectors, the HEK293T knockout clones performed similarly to their parental cell clones.

## Results

### Assessing Lentiviral Vector Titers Obtained Using HEK293 and HEK293T Cell Clones

**Figure 1:**
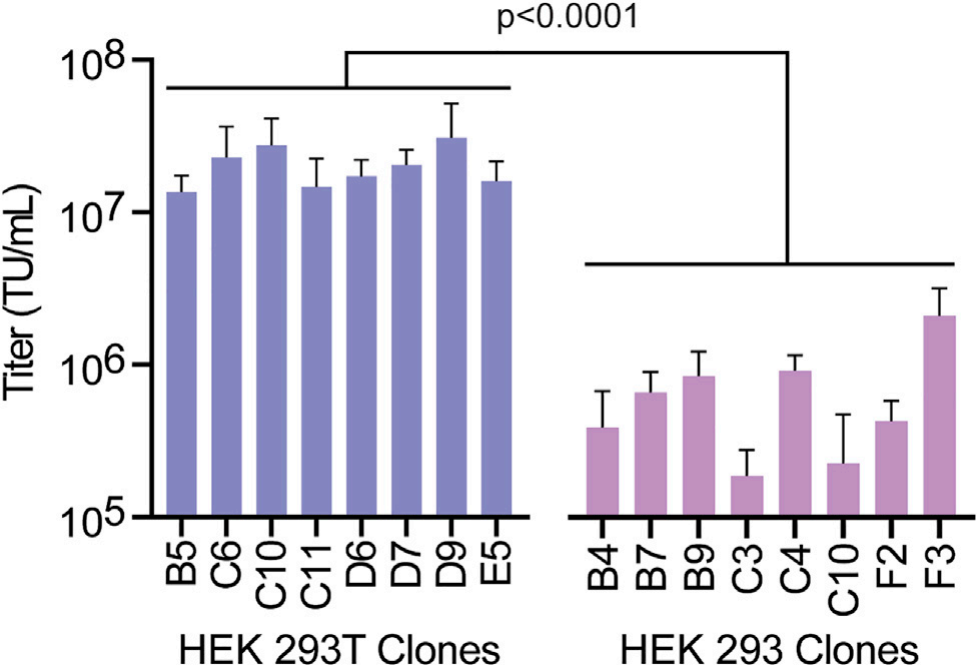
Comparison of Lentiviral Vector Titers between Individual HEK293T and HEK293 Cell Clones Four independent lots of lentiviral vectors were produced for each cell clone listed and subsequently titrated by flow cytometry. Vector titers were compared between HEK293 and HEK293T cell clones using an unpaired Student’s t test.

To determine the influence of clonal variation on lentiviral vector titers, clonal populations of HEK293 and HEK293T cells were established by limiting dilution. Clonal populations were used to generate lentiviral vector stocks by transient polyethyleneimine (PEI)-mediated transfection^12^ of a third-generation lentiviral vector system^13^ containing an EGFP-encoding lentiviral vector. Vector supernatants were titrated by transducing HEK293 cells and determining the percentage of enhanced green fluorescent protein (EGFP)-positive cells by flow cytometry.^14^ Figure 1 shows that the HEK293T clones produced an average of ∼33-fold higher lentiviral titers than did HEK293 cells. Lentiviral vector titers varied about 2-fold for HEK293T cell-derived clones and about 4-fold for HEK293 cell-derived clones. Two of the HEK293T cell clones with the highest lentiviral vector titers, referred to as D9 and C10, were used for all subsequent experiments.

### CRISPR-Cas9-Mediated Deletion of T Antigen-Encoding Sequences in HEK293T Cells

We next deleted the SV40 T antigen-encoding sequences in HEK293T cells using CRISPR-Cas9-mediated cleavage. A modified version of the pX333 plasmid^15^ encoding Cas9 and two independent, tandem U6 promoter-driven single guide RNAs (sgRNAs) targeting the beginning and the end of the T antigen-encoding sequence was used. The HEK293T D9 cell clone was subjected to nucleofection using the modified pX333 plasmid along with a GFP-encoding plasmid (pMaxGFP). Cells were subjected to flow sorting and the top 3% of the GFP-positive cells were collected individually. Sorted cell clones were expanded and genomic DNA was extracted. Two PCRs targeting the T antigen-encoding sequence and the kanamycin resistance gene (Km^R^) in the pRTAK plasmid^11^ were used to assess the presence of the T antigen-encoding and Km^R^ gene sequences in each clone. Of the 176 cell clones screened, 55 were negative using T antigen and Km^R^ sequence-specific primers, indicating that they lacked T antigen-encoding and Km^R^ gene sequences. 37 of the clones lacked a PCR signal for Km^R^ gene sequences, but T antigen-encoding sequences were detected. Three of the clones, clone #62, clone #109, and clone #126, lacked a PCR signal using T antigen-specific primers, but Km^R^ sequences were retained, indicating that these three clones contained deletions in T antigen-encoding sequences but not in Km^R^ sequences.

### Cell Clones Lacking T Antigen-Encoding Sequences Do Not Produce T Antigen Protein

**Figure 2:**
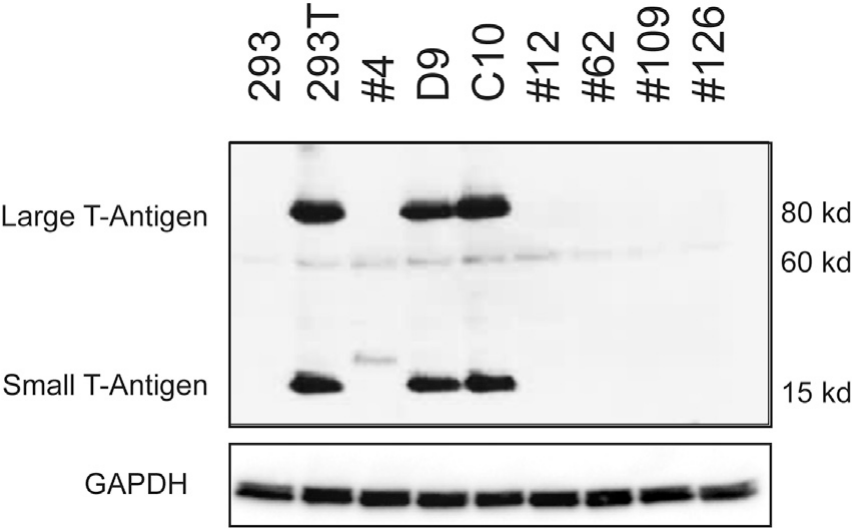
Analysis of Knockout Clones by Western Blot Extracts from bulk HEK293 and HEK293T cells, the D9 and C10 cell clones, and the #4, #12, #62, #109, and #126 deletion clones were prepared and run on a 4%–12% sodium dodecyl sulfate polyacrylamide gel. The blot was probed using an anti-SV40 T antigen monoclonal antibody (upper part) and re-probed with anti-GAPDH antibody (lower part).

We next analyzed SV40 T antigen-related proteins in cell extracts prepared from bulk HEK293 and HEK293T cells and the HEK293T cell clones D9 and C10. Cell clones #62, #109, and #126 bearing putative deletions of SV40 T antigen-encoding sequences and cell clones #4 and #12 that lacked both T antigen-encoding and Km^R^ gene sequences were also included in this analysis. An antibody targeting the 94-kDa SV40 large T antigen and the 20.5-kDa SV40 small T antigen proteins was used. The western blot analysis presented in Figure 2 shows that the expected SV40 large T antigen and small T antigen proteins were present in cell extracts derived from bulk HEK293T cells and in clone D9 and C10 cells but not in bulk HEK293 cells or the #62, #109, and #126 cell clones bearing putative deletions in the T-antigen coding regions. The T antigen-specific bands were also absent in extracts obtained using cell clones #4 and #12. Interestingly, clone #4 revealed a faint low-molecular-weight band using the PAb416 antibody. This band was not present in the other cell clones or in bulk HEK293 and HEK293T cells, and its origin is unclear.

### Analysis of T Antigen-Negative Cell Clones by NGS

**Figure 3:**
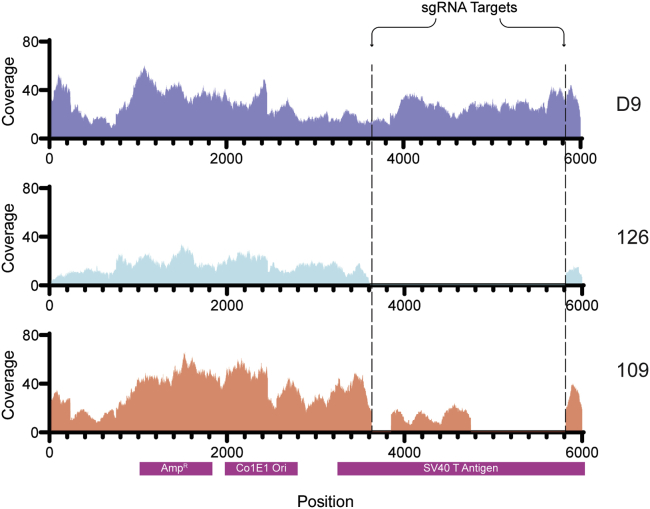
Analysis of Knockout Clones by NGS Analysis of knockout clones by NGS. Sequence coverage of pRTAK plasmid sequences in HEK293T cell clone D9 genomic DNA and in genomic DNA of the #109 and #126 deletion clones. Each individual graph shows depth coverage of pRTAK plasmid sequences at given positions along the x axis. Relevant features of the pRTAK plasmid are shown below the x axis. The vertical black lines refer to the expected Cas9 cleavage sites.

NGS was used to confirm the lack of T antigen-encoding sequences in cell clones #109 and #126. A loss of sequence coverage within the segment of T antigen sequences targeted by the CRISPR-Cas9 approach was evident (Figure 3). In the parental D9 cell clone, the T antigen-encoding sequence appeared to be intact. In the #109 cell clone, a stretch of approximately 1 kb of T antigen-encoding sequence remained, with roughly half the coverage compared to the parental D9 cell clone, indicating that the knockout was incomplete. In the #126 cell clone the entire T antigen-encoding sequence was missing, indicating full deletion of the T antigen coding region. We also confirmed that the pX333 CRISPR-Cas9 plasmid sequence used to remove the T antigen-encoding sequences was not retained in clones #109 and #126 (B.I., unpublished data).

### Analysis of T Antigen-Negative Cell Clones by TLA

**Figure 4:**
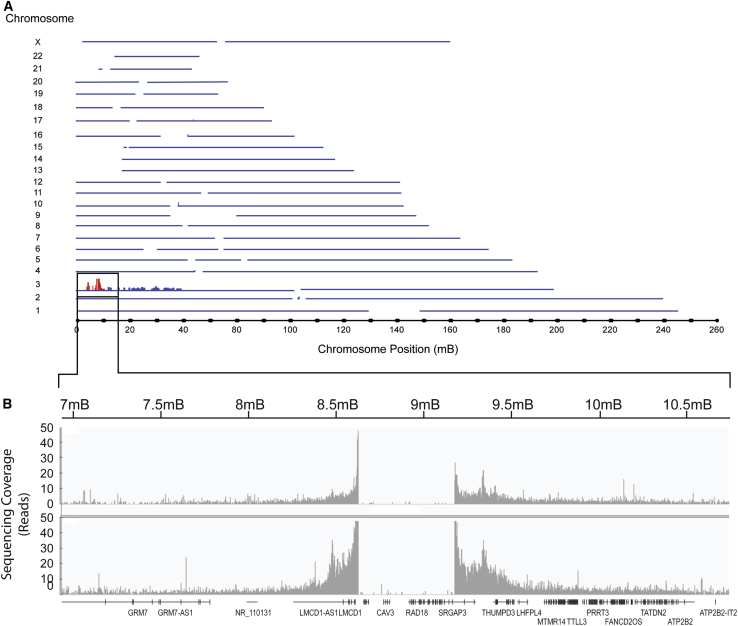
Analysis of Knockout Clones by Targeted Sequencing (A) Targeted sequencing of T antigen integration sites. The plot shows TLA sequence coverage across the HEK293T cell clone D9 genome using primers targeting the pRTAK plasmid origin of replication. The single plasmid integration site present on chromosome 3 (chr3) of the HEK293T D9 cell clone is shown in red. (B) TLA sequence coverage of the plasmid integration site referred to in (A). The x axis shows genomic features from human chr3: 6,938,850–10,764,483. The two boxplots with gray bars indicate sequence coverage observed when enrichment was conducted with primers targeting the origin of replication (upper boxplot) or T antigen-encoding sequences (lower boxplot). The y axis is limited to 50-fold coverage. Data in this figure are from the parental D9 cell clone, but they are representative of deletion clones #109 and #126, as they yielded similar integration sites. Box magnified area is not to scale.

TLA sequencing was used to further characterize the integration sites of the T antigen-encoding pRTAK plasmid in clones D9, #109, and #126. A single plasmid integration site on chromosome 3 was identified (Figure 4A), which spanned positions 8632193 through 9182543 of the hg19 human reference genome, with plasmid junctions at positions 235 and 2459, respectively. These integration sites were the same as those identified through whole-genome sequencing. Interestingly, the TLA sequencing also identified a loss of native DNA sequence of approximately 550 kb between these junctions. This deleted region contains the *SSUH2*, *CAV3*, and *RAD18* gene sequences and exons 2–22 of the *SRGAP3* gene (Figure 4B). Elevated coverage of the integrated plasmid sequence relative to adjacent genomic DNA sequences suggests that there are multiple plasmid copies at this locus. Clone #126, which showed a complete removal of the T antigen-encoding sequence, also lacked an additional 1,950 bp of genomic sequence, with plasmid-genome junctions on chromosome 3 at positions 8630243 and 9182543 (B.I., unpublished data).

### Effects of Removal of T Antigen-Encoding Sequences on Lentiviral or AAV Vector Titers

**Figure 5:**
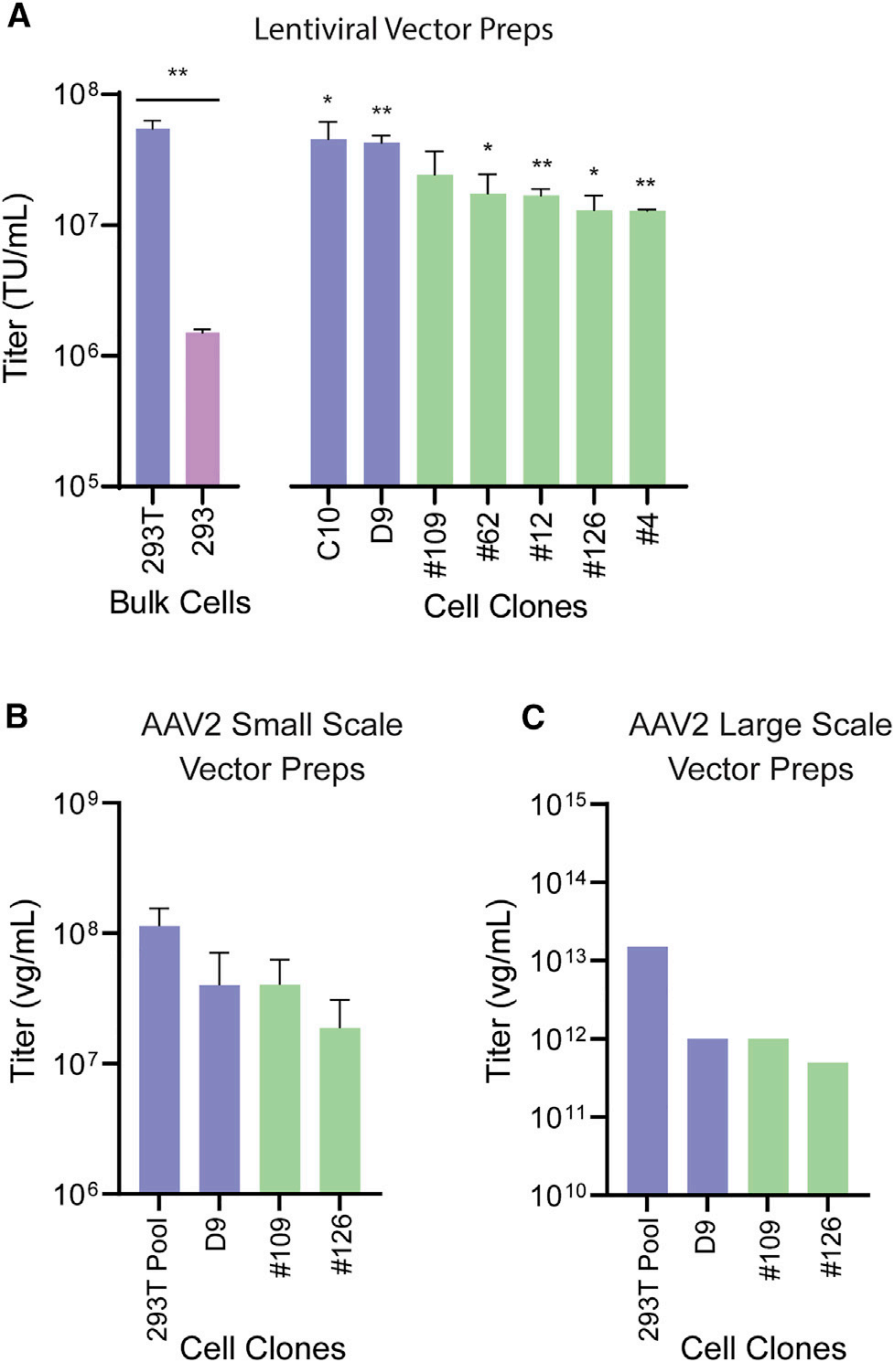
Vector Production Using Knockout Clones Lentiviral vectors were produced by PEI-mediated transfection using a third-generation lentiviral vector system involving an EGFP-encoding vector plasmid. The vector-containing supernatants were harvested at 72 h. Vector aliquots were titrated by transduction of HEK293 cells. 293T and 293 refer to bulk HEK293T and HEK293 cells, respectively. C10, D9, #109, #62, #12, #126, and #4 refer to cell clones. Functional titers were determined by FACS analysis. Error bars represent means ± standard error of two or three independent experiments, and statistical analysis was performed using an unpaired student's T test. (B and C) AAV2 vectors were produced by transient transfection of a polyclonal 293T cell pool​^27^ using the pAAV2-NLS-GFP and pAAV2 RepCap plasmids, and the Ad helper plasmid 449B. This was performed at small scale (B) and large scale (C). The cells were collected 48 h later and freeze-thaw lysates were prepared. Vector DNA copies (vector genomes [vg]/mL) in the lysate were determined by qPCR using primers for the CMV promoter sequence. Error bars represent means ± standard error of four independent experiments.

We next investigated whether T antigen knockout clones exhibited altered vector production capacity compared to HEK293T cells. Lentiviral vectors were produced using the HEK293T D9 and C10 cell clones, the #4 and #12 deletion clones lacking T antigen and Km^R^ gene sequences, and the T antigen deletion clones #62, #109, and #126. A third-generation lentiviral vector system involving the pNL(CMV)EGFP/CMV/WPREDU3 vector plasmid was used.^13^ Encouragingly, we observed that deletion of the T cell antigen coding region did not substantially impair lentiviral vector production capacity (Figure 5A). Lentiviral vector titers from T antigen knockout clones were on average 30% of those obtained from HEK293T cell clones D9 and C10 but were still >10-fold higher than those obtained with bulk HEK293 cells.

We also tested the impact of T antigen-encoding sequences on AAV vector production. We prepared small-scale AAV2 vector stocks (n = 4) (Figure 5B) and one large-scale AAV2 vector stock (Figure 5C) using clone D9 and the #109 and #126 deletion clones. Vector genome copies were determined by qPCR.^16^ Figure 5B shows that AAV2 genome-based vector titers for small-scale stocks were similar between clone D9 cells and clone #109 cells, while for clone #126 cells the titers were about half of those observed with D9 cells. For the large-scale vector stocks (Figure 5C), a similar pattern was observed compared to the small-scale AAV2 vector preparation.

In addition, we compared AAV-DJ vector production involving bulk HEK293 and HEK293T cells, clone D9 and C10 cells, and the T antigen-negative cell clones #4, #12, #62, #109, and #126. Overall, the AAV-DJ vector titers observed were very similar (M.M., unpublished data).

## Discussion

Current manufacturing strategies for lentiviral vector production commonly involve transient transfection of HEK293T cells. HEK293T cells have been repeatedly shown to produce superior vector titers compared to HEK293 cells.^2^ Furthermore, HEK293T cells have more advantageous growth kinetics and transfection efficiencies than do HEK293 cells.^1^^,^^17^^,^^18^ However, the use of the HEK293T cell line to manufacture vectors for *in vivo* applications raises safety concerns due to the presence of oncogenic SV40 T antigen-encoding sequences. In addition, the HEK293 cell line and its derivatives (e.g., HEK293T cells) harbor five to six copies of Ad5 DNA,^6^ and process-related impurities involving Ad5 sequences encoding E1A/E1B proteins need to be accounted for to assure product safety.

According to US Food and Drug Administration (FDA) guidance for human gene therapy investigational new drug applications (https://www.fda.gov/regulatory-information/search-fda-guidance-documents/chemistry-manufacturing-and-control-cmc-information-human-gene-therapy-investigational-new-drug), if cells with a tumorigenic phenotype, such as HEK293T cells, are used for viral vector production, residual substrate DNA must be limited to assure product safety. In addition to controlling host cell DNA content, the level of transforming sequences in clinical products should be tightly controlled to limit patient exposure. Clinical products produced in HEK293T cells will require testing for residual adenovirus E1 and SV40 large T antigen sequences. This is particularly true with AAV vectors, which can package large amounts of non-vector DNA (e.g., cellular DNA).

Merten et al.^1^ used qPCR to determine residual adenovirus E1A and SV40 large T antigen-encoding sequences in concentrated lentiviral vector samples. These studies revealed between 0.37 × 10^5^ and 2.4 × 10^5^ copies of E1A sequences and between 0.69 × 10^5^ and 4.2 × 10^5^ copies of SV40 large T antigen-encoding sequences per milliliter of the purified vector sample. The PCR amplicons observed were small because vector samples had been pretreated with Benzonase, indicating that they would not have the capacity to encode functional E1A and T antigen proteins. To evaluate the impact of residual E1A and SV40 large T antigen-encoding sequences in vector samples, Merten et al.^1^ determined the extent at which such sequences could be passively transferred to target cells upon transduction and amplification in C8166-45 cells. One week after transduction, E1A and SV40 large T antigen-encoding sequences were found to be below the level of detection of the assay used. The authors concluded that the small amounts of residual E1A and SV40 T antigen-encoding sequences detected in the purified vector samples would likely be insufficient to transform target cells and that the risks posed by the residual E1A and SV40 T antigen-encoding sequences are low, particularly in the context of *ex vivo* vector administrations.

In this study we deleted the SV40 T antigen-encoding sequences in HEK293T cells using a CRISPR-Cas9 approach and determined the impact on lentiviral vector and AAV vector titers. The interaction of the large T antigen with SV40 origins of replication present in lentiviral packaging plasmids has long been postulated to underlie the increased lentiviral productivity in HEK293T cells.^5^ The lentiviral vector plasmid used in this study contains an SV40ori sequence. However, our work has revealed that the removal of the T antigen-encoding sequence only marginally reduced lentiviral titers, supporting the notion that additional cellular factors^17^ may be responsible for the boost in vector titers relative to HEK293 cells. Our findings support recent studies involving RNAi-mediated knockdown of SV40 T antigen-encoding sequences. These studies also observed no significant effect on vector production yields.^19^

While the results of the NGS, TLA, and the western blot analyses performed on the T antigen deletion clones support our assumption that the lack of T antigen-encoding sequences does not impact the vector production capacity of such cells, the deletion clones were found to grow slower compared to HEK293T cells with a growth profile similar to HEK293 cells. There may be numerous additional differences^6^ that could account for the observation. This will necessitate further research in the future.

In summary, the production of clinical-grade lentiviral vectors requires substantial modification of the techniques used for producing research-grade lentiviral vectors. The most important of these modifications center around producing vectors free of process- and product-derived impurities. In this work, we engineered the commonly used HEK293T cell line to eliminate SV40 T antigen-related impurities. Overall, these modifications did not compromise the utility of the cell line for vector production. However, as shown in Figure 5A, lentiviral vector titers using cells derived from the #62, #109 and #126 T antigen deletion clones were reduced compared to the C10 and D9 cell clones and bulk HEK293T cells bearing intact T antigen-encoding sequences. This may limit the usefulness of these deletion clones in the context of large-scale commercial manufacturing settings. Optimizing the upstream manufacturing steps may help alleviate these problems.

## Materials and Methods

### Plasmid Constructs

The pX333 plasmid^15^ was used to express two independent sgRNAs targeting SV40 T antigen-encoding sequences. This plasmid was a gift from Andrea Ventura (Addgene plasmid #64073; http://addgene.org/64073; RRID:Addgene_64073). The sgRNAs targeting the beginning and end of the T antigen-encoding sequence present in the pRTAK plasmid that was used originally to produce the HEK293T cell line^11^ were designed using CRISPRdirect (https://crispr.dbcls.jp/).^20^ Potential off-target editing sites in the human genome were evaluated using the Off-Spotter tool (https://cm.jefferson.edu/Off-Spotter/). Two sgRNA-encoding synthetic DNA fragments targeting the end and the beginning of the T antigen-encoding sequence, respectively, were obtained by annealing the T antigen end forward (Fwd) (5′-CACCGCAGCCATATCACATTTGTAG-3′) and T antigen end reverse (Rev) (5′-AAACCTACAAATGTGATATGGCTGC-3′) oligonucleotides and the T antigen beginning Fwd (5′-CACCGTATGCTCATCAACCTGACTT-3′) and T antigen beginning Rev (5′-AAACAAGTCAGGTTGATGAGCATAC-3′) oligonucleotides. The annealed sequences were sequentially cloned into pX333 using BbsI and BsaI (New England Biolabs, Ipswich, MA, USA) and T4 DNA ligase (Thermo Fisher Scientific, Waltham, MA, USA).

### Cell Culture

HEK293T cells (CRL-3216, ATCC, Manassas, VA, USA) and HEK293 cells (catalog no. 103, NIH AIDS Reagent Program, Germantown, MD, USA) were grown in Dulbecco’s modified Eagle’s medium (DMEM) containing high glucose (4.5 g/L), 2 mM l-glutamine, 10% heat-inactivated fetal bovine serum (FBS), 100 U/mL penicillin, and 100 μg/mL streptomycin. Cell culture reagents were purchased from Gibco (Invitrogen, Carlsbad, CA, USA). CHO-K1 cells (CCL-61, ATCC, Manassas, VA, USA) were grown in Roswell Park Memorial Institute (RPMI) 1640 medium containing 2 mM l-glutamine, 10% heat-inactivated FBS, 100 U/mL penicillin, and 100 μg/mL streptomycin. Cell culture reagents were purchased from Gibco (Invitrogen, Carlsbad, CA, USA)

### Cell Cloning

HEK293T and HEK293 cells were cloned by limiting dilution into 96-well plates. Wells containing single cells were identified by microscopy. Selected clones were expanded in six-well plates and then transferred to T75 cell culture flasks.

### CRISPR-Cas9-Mediated Gene Knockout and Analysis of Clones

CRISPR-Cas9-mediated gene knockout was carried out by nucleofection, using a Cell Line Nucleofector kit V (Lonza, Basel, Switzerland) according to the manufacturer’s instructions. Briefly, 1.2 × 10^6^ HEK293T clone D9 cells were nucleofected using 500 ng of the provided pMaxGFP plasmid and 5 μg of the modified pX333 plasmid encoding two sgRNAs targeting the SV40 large T antigen sequence. Cells were incubated in DMEM/10% FBS for 36 h, with a complete replacement of the medium the morning after nucleofection. The top 3% of GFP-expressing cells were sorted into 96-well plates, using a BD FACSAria III cell sorter (Becton Dickinson, Franklin Lakes, NJ, USA). Cell clones were expanded to 24-well plates. Genomic DNA was extracted using a DNeasy Blood & Tissue kit (QIAGEN, Hilden, Germany). Clones were screened by PCR, using a primer set targeting the large T antigen-encoding sequence (Fwd, 5′-ATGCCTGTTTCATGCCCTGA-3′, Rev, 5′-GGGGAGTCCAGAGATTTGCC-3′) and a primer set targeting the Km^R^ gene sequence (Fwd, 5′-CCGGTGCCCTGAATGAACTG-3′, Rev, 5′-CGGGTAGCCAACGCTATGTC-3′). PCR reactions were carried out using Taq DNA polymerase, recombinant (Invitrogen, Carlsbad, CA, USA), with an initial denaturation step of 5 min at 95°C, followed by 35 cycles of denaturation (30 s at 95°C), annealing (30 s at 52°C), and extension (90 s at 72°C), as well as a final extension step of 7 min at 72°C.

### Western Blot Analysis of Cell Clones

Cell aliquots (corresponding to 2 × 10^6^ cells) were lysed on ice using radioimmunoprecipitation assay (RIPA) buffer (Sigma-Aldrich, St. Louis, MO, USA) containing a protease inhibitor cocktail (Roche Diagnostics, Manheim, Germany). For SDS-PAGE, cell lysates containing approximately 50 μg of total cellular protein per sample were denatured by treating with SDS loading buffer containing a reducing agent. The reduced protein samples were run on a NuPAGE 4%–12% Bis-Tris SDS polyacrylamide gel (Invitrogen/Life Technologies, Carlsbad, CA, USA) at 150 V for 50 min in 4-morpholinepropanesulfonic acid (MOPS)-SDS running buffer. The proteins were transferred to a polyvinylidene fluoride (PVDF) blotting membrane (Invitrogen/Life Technologies), and the blot was incubated in a 5% dry milk solution for 1 h. The blot was then incubated with an anti-SV40 T antigen monoclonal antibody (PAb416; Thermo Fisher Scientific, Waltham, MA, USA). Finally, the blot was washed and probed using goat anti-mouse immunoglobulin G (IgG) linked to horseradish peroxidase using the SuperSignal West Dura substrate (Thermo Fisher Scientific, Rockford, IL, USA). The blot was stripped in Restore western blot stripping buffer (Thermo Scientific, Rockford, IL, USA), according to the manufacturer’s instructions, and re-probed with anti-GAPDH antibody (mAbcam 9484; Abcam, Cambridge, MA, USA). To visualize the bands, the membrane was exposed to BioMax MR film (Carestream Health, Rochester, NY, USA) for 30 s.

### Whole-Genome Sequencing Analysis of Cell Clones

Genomic DNA was extracted from the HEK293T D9 cell clone and the #109 and #126 deletion clones using a DNeasy Blood & Tissue extraction kit (QIAGEN). Approximately 3 μg of genomic DNA per sample was submitted to Genewiz (South Plainfield, NJ, USA), where NGS library preparation, sequencing, and de-multiplexing were conducted. Briefly, genomic DNA for each sample was fragmented and 200- to 500-bp fragments were selected and sequencing adapters were ligated. Libraries were sequenced on an Illumina HiSeq platform using high-output mode with a 2× 150-bp paired-end (PE) sequencing configuration. Approximately 400 million paired-end reads were generated for each sample. All samples yielded greater than 30-fold coverage when mapped against a reference genome (hg38) using Isaac.^21^ Reads were mapped against the pRSV1609^6^ and pX333 sequence using Hexagon. Integration sites were identified using DI profiler.^22^, ^23^, ^24^, ^25^

### TLA Sequencing

TLA sequencing^9^ was performed by Cergentis (Utrecht, The Netherlands). For TLA, two primer sets were used: one targeting the plasmid origin, 5′-GCAGATTACGCGCAGAAA-3′ and 5′-TTTGTGATGCTCGTCAGG-3′, and one targeting the T antigen-encoding sequence, 5′-CGAAGCAGTAACAATCAACC-3′ and 5′-CTGCTGACTCTCAACATTCT-3′. Reads were mapped using Burrows-Wheeler aligner’s Smith-Waterman alignment (BWA-SW).^22^ Reads were aligned to both the human genome (hg19) and to the putative sequence of the T antigen-encoding pRTAK plasmid.^6^

### Lentiviral Vector Production

Lentiviral vector production was carried out by transient transfection using 15-cm plates (Nunc, Thermo Fisher Scientific, Waltham, MA, USA) as described.^12^, ^13^, ^14^ The transfections involved 8 μg of pNL(CMV)EGFP/CMV/WPREΔU3 DNA, 6.4 μg of pCD/NL-BHΔ1 DNA, 8.32 μg of pCMV-rev DNA, and 2.72 μg of pCEF-VSV-G DNA per 15-cm plate. The vector-containing supernatants were harvested ∼60 h post-transfection, centrifuged at 500 × *g* for 5 min, and filtered through a 0.45-μm filter cup (Corning Life Sciences, Corning, NY, USA). Vector supernatants were titrated by transduction of HEK293 cells as previously described.^26^ Functional titers were determined by fluorescence-activated cell sorting (FACS) analysis on a BD FACSCanto II flow cytometer.

### Production of Recombinant AAV2 Vectors

HEK293T cell pools and the HEK293T-derived D9, #109, and #126 cell clones were cultured in DMEM supplemented with 10% FBS (HyClone, Logan, UT, USA), 2 mM l-glutamine, 100 U of penicillin/mL, and 0.1 mg of streptomycin/mL (Thermo Fisher Scientific).

Recombinant AAV2 vectors expressing a nuclear localized GFP were produced as previously described.^27^ Briefly, cells were co-transfected using the pAAV2-NLS-GFP, pAAV2 RepCap, and the Ad helper plasmid 449B.^16^

For large-scale AAV2 vector production, cells were plated in 150-cm dishes and 48 h after transfection they were harvested by scraping. The collected cells were centrifuged at low speed and lysed in 1× TD buffer (140 mM NaCl, 5 mM KCl, 0.7 mM K_2_HPO_4_, 25 mM Tris-HCl [pH 7.4]) containing 0.5% deoxycholate (sodium deoxycholate, Sigma) and Benzonase (Sigma, ultrapure 25,000 U), and incubated for 1 h at 37 °C. Recombinant (rAAV-GFP) vectors were purified by cesium chloride gradient centrifugation. DNase-resistant genome copy numbers for the vectors were determined by qPCR using the TaqMan system (Applied Biosystems) with primers specific to the CMV promoter contained within the packaged genome. Briefly, 1-μL aliquots of a 1:1,000 dilution in double-distilled water were used. The primer sequences used are as follows: CMV promoter, forward, 5′-CATCTACGTATTAGTCATCGCTATTACCAT-3′; reverse, 5′-TGGAAATCCCCGTGAGTCA-3′.

For small-scale AAV2 vector production, cells were co-transfected with the pAAV2-NLS-GFP and pAAV2 RepCap plasmids, and the Ad helper plasmid 449B using 10-cm plates. Cells were harvested 48 h after transfection. Cell pellets were resuspended in 500 μL of 1× TD buffer and the cell lysates were frozen/thawed three times. Cellular debris were removed by centrifugation at 1,100 rpm at room temperature. 100-μL aliquots of the supernatants were treated with Benzonase (1 h at 37°C), followed by phenol/chloroform extraction. Vector DNA copies in the lysate were determined by qPCR using primers for the CMV promoter sequence as stated above.

## Author Contributions

A.K. and J.R. designed the study. D.H.B., M.M., B.I., A.K., S.A., T.A., and J.M. performed the experiments. A.K., B.I., S.F., M.M., A.E.T., J.A.C., and J.R. analyzed the data. J.R., B.I., and A.E.T. wrote the manuscript. All authors reviewed the manuscript.

## Conflicts of Interest

The authors declare no competing interests.
